# Randomized Controlled Trials of Weight Loss Before Hernia Surgery: A Systematic Review and Meta-Analysis

**DOI:** 10.3389/jaws.2025.15234

**Published:** 2025-10-13

**Authors:** Nadia McLurcan, David L. Sanders, Marianne Hollyman, Sarah E. Lamb, John M. Findlay

**Affiliations:** ^1^ Academic Department of Abdominal Wall and Upper Gastrointestinal Surgery, North Devon District Hospital, Royal Devon University Healthcare NHS Foundation Trust, Barnstaple, United Kingdom; ^2^ NIHR Exeter Biomedical Research Centre, University of Exeter Medical School, Exeter, United Kingdom; ^3^ Department of Upper Gastrointestinal Surgery, Musgrove Park Hospital, Taunton, United Kingdom; ^4^ Department of Public Health and Sports Sciences, University of Exeter Medical School, Exeter, United Kingdom; ^5^ Department of Clinical and Biomedical Sciences, University of Exeter Medical School, Exeter, United Kingdom

**Keywords:** hernia, weight loss, preoperative optimization, prehabilitation, complications

## Abstract

**Purpose:**

Hernia repairs are one of the commonest operations performed in the world. In Europe and the United States of America more than half of patients undergoing hernia surgery are overweight or obese; body weight is therefore key modifiable risk factor before surgery. We aimed to identify, appraise, and synthesise the randomized controlled trial (RCT) evidence for any weight loss interventions before any form of hernia surgery.

**Methods:**

A search was performed in April 2025 of the PubMed, EMBASE and CENTRAL databases. Meta-analysis was performed using random effects to assess mean difference in weight, and fixed effects for odds ratio of complications. Bias was assessed using the Cochrane Rob2 tool. Certainty effect was assessed using GRADE methodology. This review was registered with PROSPERO (ID 1024784).

**Results:**

1,707 studies were screened, 20 retrieved, and 4 reports of 3 RCTs included. A total population of 219 patients largely underwent ventral hernia repair, although 2 RCTs also included patients undergoing non-hernia surgery. Interventions comprised very low calorie diets (VLCD), and a multidisciplinary programme. Meta-analysis for VLCD did not show a reduction in weight loss (3.64 kg [95% confidence interval −2.07 – 9.35]) or complications [odds ratio 0.36 (0.1–1.28)]. Risk of bias was “high,” and certainty of effect “very low.”

**Discussion:**

Despite the prevalence and importance of obesity in patients undergoing elective hernia repair, and the popularity of weight loss as part of prehabilitation, the randomized evidence of how to effect weight loss, and whether this translates into an improvement in clinical outcomes is minimal. This highlights the urgent need for large and robust RCTs to determine if weight loss before hernia repair is effective in improving outcomes for patients, and how this is best achieved.

## Introduction

Hernias are one of the commonest conditions globally, with prevalence projected to continue increasing [1]. The majority of these comprise groin hernias (largely inguinal) and ventral abdominal hernias [1]. Hernias can significantly affect patients’ quality of life (QoL) as well as risking emergency complications, and so hernia repair is one of the commonest operations performed across the world, thought to be well in excess of 20 million operations per year [2]. Most patients with hernias are in their sixth decade [3], and so surgery is often performed in the presence of other medical conditions.

Outcomes from hernia surgery are heavily influenced by a number of factors, beyond technical considerations of approach to the operation. Several patient factors can be modified, thereby providing the chance to “pre-optimise” these in a window before elective surgery, thereby reducing some of this risk [4]. The primary such risk factors are body weight, smoking and diabetic control [4].

Of these, the effect of body weight on operative outcomes has been most commonly studied. Most patients undergoing hernia surgery are overweight or obese, and the impact of this on the results of surgery seems profound, in particular the risk of hernia recurrence and complications (during and after surgery), with worse subsequent QoL [5]. In ventral hernias, obesity (body mass index [BMI] >30) significantly increases of both, with multivariate effect sizes (odds ratios) of between 1.5 and >3 depending on BMI class demonstrated by large national database analysis and systematic review, effects which are compounded by additional operative risk factors such as complexity [6, 7]. Similar results have been demonstrated for inguinal hernia surgery [5, 8]. Most patients undergoing ventral hernia surgery are obese, and nearly half of those undergoing inguinal hernia surgery were overweight [6, 8].

Obesity is therefore a crucial modifiable risk factor, and weight management is strongly recommended during a period of “pre-optimisation” in the window available before surgery [4].

However, hernias often limit patients’ ability to lose weight due to their functional and psychological impact, and so only a very small minority of patients are able to lose significant weight (defined as 10%) before IHR [6]. These patients are therefore at significantly greater risk of complications, hernia recurrence, a worse functional outcome and QoL gains after. Whilst advisable, the realities of this are unclear, with a number of unanswered questions. The aim of this review was to systematically identify and appraise the randomized controlled trial (RCT) literature of weight loss interventions before hernia surgery.

## Methods

### Literature Search

A final literature search was performed of the PubMed^1^, EMBASE^2^ and CENTRAL^3^ databases on 2nd April 2025 in accordance with the Preferred Reporting Items for Systematic Reviews and Meta-Analyses [9] using the following search term: (weight OR obes* OR preoptimi* OR prehabilit* OR BMI OR “Body Mass Index” OR diet OR exercise) and (hernia OR abdominal wall reconstruction) AND (randomized). Bibliographies of retrieved articles were searched. Studies were screened on the basis of title and abstract, and reviewed independently by two authors (NM and JMF). This review was registered with PROSPERO (ID 1024784).

### Inclusion and Exclusion Criteria

We included randomized controlled trials (RCT) assessing any form of intervention to induce weight loss before hernia surgery, compared with any control. We excluded conference abstracts and study protocols.

### Endpoints

Primary endpoint was the effect of interventions on weight (absolute or any other metric of obesity). Secondary endpoints were any effects on operations, their results or complications.

### Data Collection

Data were collected independently by two authors (JMF and NM). In addition to the above endpoints, data comprised study design, population, intervention and control.

### Assessment of Study Bias

Bias was assessed using RoB 2: A revised Cochrane risk-of-bias tool for randomized trials, for studies and outcomes [10].

### Meta-Analysis

Meta-analysis was performed using Cochrane RevMan 8.13.0, for studies reporting and measuring endpoints similarly. Heterogeneity was assessed using Restricted Maximum-Likelihood (REML); random effects models with inverse variance were used if I^2^ >50%; fixed effects models were used with inverse variance if I^2^ <50%). Neither sensitivity nor assessment of publication bias were undertaken as these would not be meaningful for analyses of just 2 studies.

### Certainty of Effect

Certainty of effect was determined using GRADE methodology [11].

## Results

### Literature Search

**FIGURE 1:**
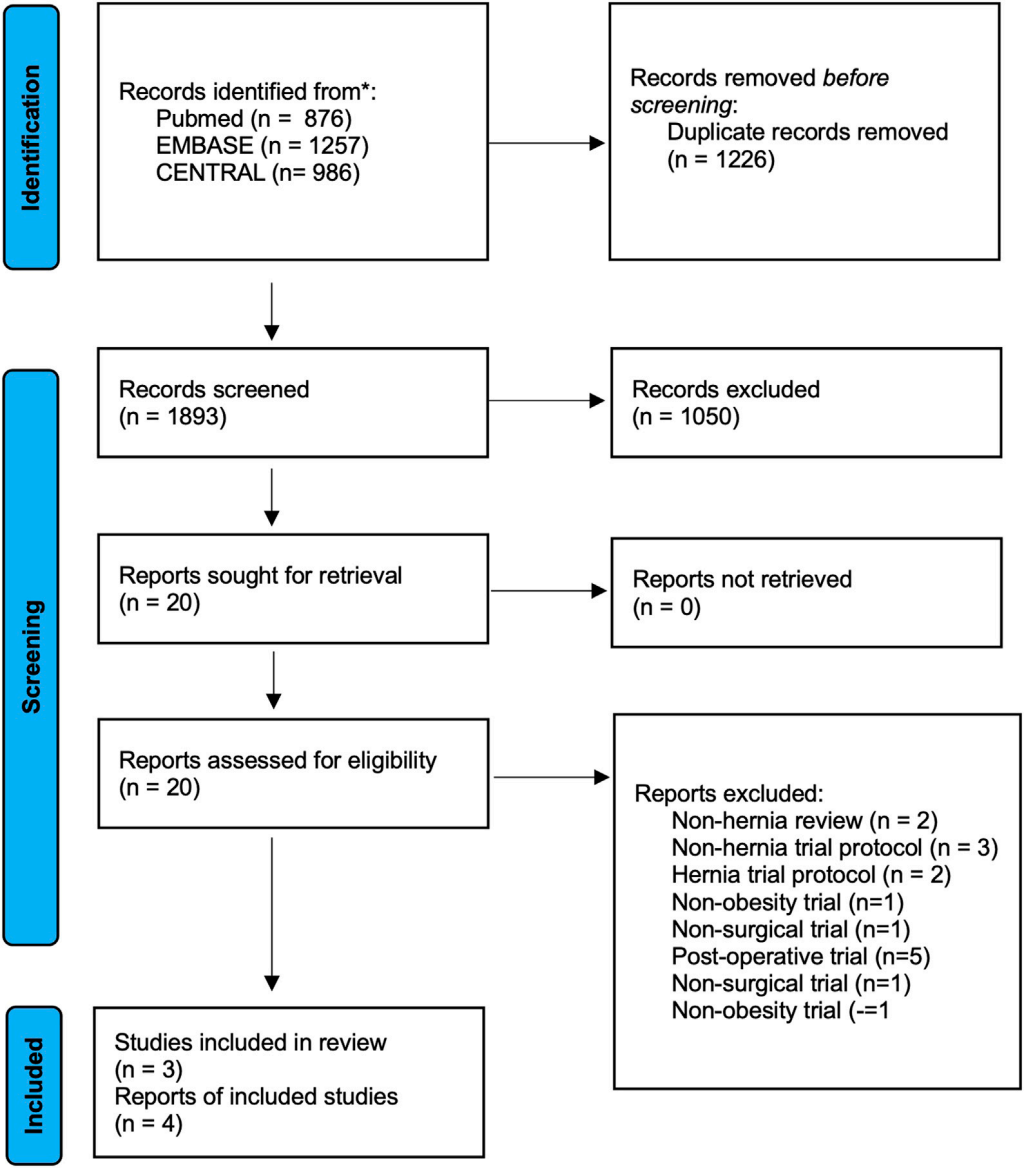
PRISMA diagram.

3,119 studies were identified. After exclusion of duplicates 1,707 studies were screened, 20 studies retrieved, and 4 included (Figure 1).

### Included Studies

We included 4 reports in total of 3 RCTs (Table 1). Their populations comprised patients with a BMI >30, awaiting ventral hernia repair [12, 13], or as part of a mixed cohort with laparoscopic cholecystectomy and gynaecological surgery [14], and also inguinal hernia repair [15]. Their interventions comprised a very low calorie diet (VLCD) in 2 RCTs [14, 15], or a multidisciplinary (non-pharmacological) programme [12, 13]. Their controls comprised standard care [14], healthy eating information [15] or non-specialist counselling [12, 13]. Primary outcomes were feasibility in 2 [14, 15], and being hernia and complication free at 30 days [13] and 2 years [12].

**TABLE 1:** Included studies.

Study	Population	Intervention	Comparison	Outcomes of intervention
Griffin et al, British Journal of Nutrition 2024	51 adults with BMI >30, awaiting ventral hernia (n = 8), laparoscopic cholecystectomy and gynaecological surgery	VLCD for 2–12 weeks pre-operatively	No dietary intervention or advice	Primary: Feasibility (acceptable, adherence). Secondary: greater weight loss, better QoL, similar 30 days complications
Hollis et al, Nutrition and Dietetics 2020	50 adults, BMI >30, awaiting ventral or inguinal (n = 19) or laparoscopic cholecystectomy surgery	VLCD for 8 weeks pre-operatively	Healthy eating information	Primary: Feasibility; Secondary: greater weight loss, better QoL, similar complications
Bernardi et al, 2022	108 patients, from 118 adults (BMI 30–40) awaiting ventral hernia repair	Prehabilitation programme (up until 7% weight loss or 6 months)	Standard counselling (up until 7% weight loss or 6 months)	Primary: No difference in complications, or being hernia and complication-free at 2 years
Liang et al, 2018	118 adults (BMI 30–40) awaiting ventral hernia repair	Prehabilitation programme (up until 7% weight loss or 6 months)	Standard counselling (up until 7% weight loss or 6 months)	Primary: More likely to be hernia and complication-free at 1 month Secondary: Similar weight loss, but more drop-outs and emergency repairs

BMI, body mass index; VLCD, very low calorie diet.

One RCT (Liang et al. [13]) described a multidisciplinary prehabilitation programme in patients awaiting ventral hernia repair, at a single centre in the USA [13]. The programme comprised an intensive programme (multidisciplinary consultation, goal-directed intensive monitoring, and peer support) and was compared with standard counselling with monthly assessment. The intervention and control were both endpoint and time limited, in that patients proceeded to surgery if they met the predefined 7% of body weight loss target, or had undertaken the intervention or control for 6 months without gaining weight. The population comprised 118 patients with a BMI 30–40 awaiting ventral hernia surgery (for a hernia between 3 and 20 cm width), and was undertaken in a single centre in the United States of America (United States). Assessors were blinded where possible. Randomization was stratified by BMI.

The initial report described the outcomes at 1 month (primary: being hernia and complication-free; and secondary: complications, change in weight and obesity parameters) [13]. Four patients dropped out (3 from prehabilitation group), and four required emergency repair (within the prehabilitation group), although there were no statistical differences between the two groups. 78 patients (66%) proceeded to elective surgery. There were no significant differences in total weight loss, parameters such as hip and waist size, and whether patients met the 7% weight loss target. However, more patients proceeded to elective surgery (81.5 -v- 58.6%; p = 0.013) and were hernia and complication-free at 1 month (69.5% -v- 47.5%; p = 0.015) in the prehabilitation group.

The 2 years follow up report of 108/118 patients, found no differences in major complications (10.2% vs. 9.1%, P = 0.438) and hernia and complication-free status (72.9% vs. 66.1%, P = 0.424) [12].

Two other RCTs assessed the impact of very low calorie diets (VLCDs) in two mixed populations. Hollis et al described an RCT of 46 patients with a BMI >30 awaiting ventral or inguinal hernia repair, or laparoscopic cholecystectomy, at a single centre in Australia [15]. The intervention was an 8 weeks bespoke VLCD, comprising 700–800 calories with 0.75 mg/kg protein; this was delivered using meal replacement shakes and salads, and was supported by one-to-one dietetic advice to promote adherence and manage symptoms. The control group received standard written information regarding weight loss before surgery. Primary outcomes were feasibility metrics; secondary outcomes were surgery complications at 30 days, QoL and anthropometrics. 50 patients were randomised; 2 patients dropped out from each arm. Patients in the intervention group lost more weight (mean −6.6 kg), had a greater reduction in waist circumference and fat mass, did not lose lean muscle mass, and had better QoL (HrQOL). There were no differences in surgical complications.

Griffin et al, described a similar RCT, in a separate centre in Australia [3]. 52 patients with a BMI >30, awaiting ventral (but not inguinal) hernia repair, laparoscopic cholecystectomy or major gynaecological surgery were randomized to either a VLCD or control for 2–12 weeks before surgery. The VLCD was similar to that used by Hollis et al; the control group received no advice or intervention. Beyond the primary outcomes of feasibility, patients in the intervention arm lost more weight and fat, with greater reduction in waist circumference. There were no differences in surgical complications at 30 days.

### Meta-Analysis

**FIGURE 2:**
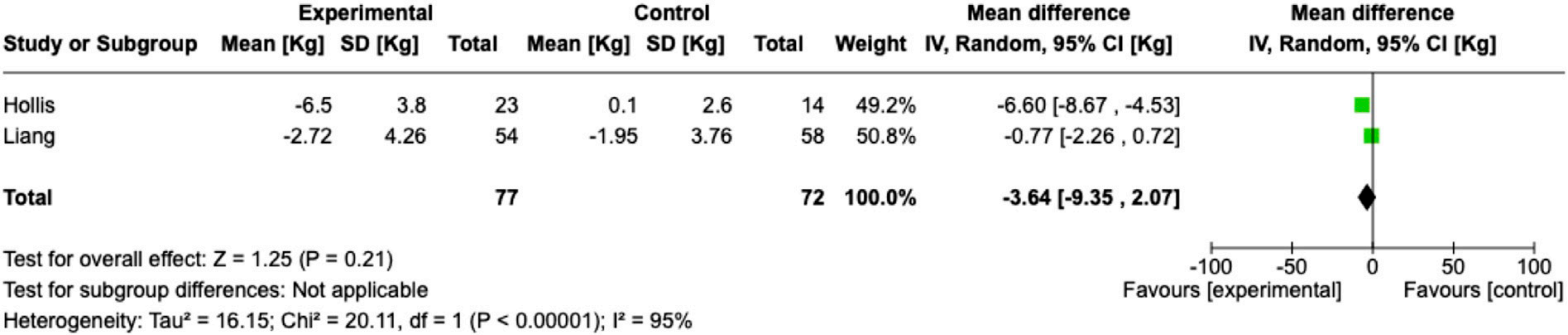
Meta-analysis of weight loss.

**FIGURE 3:**
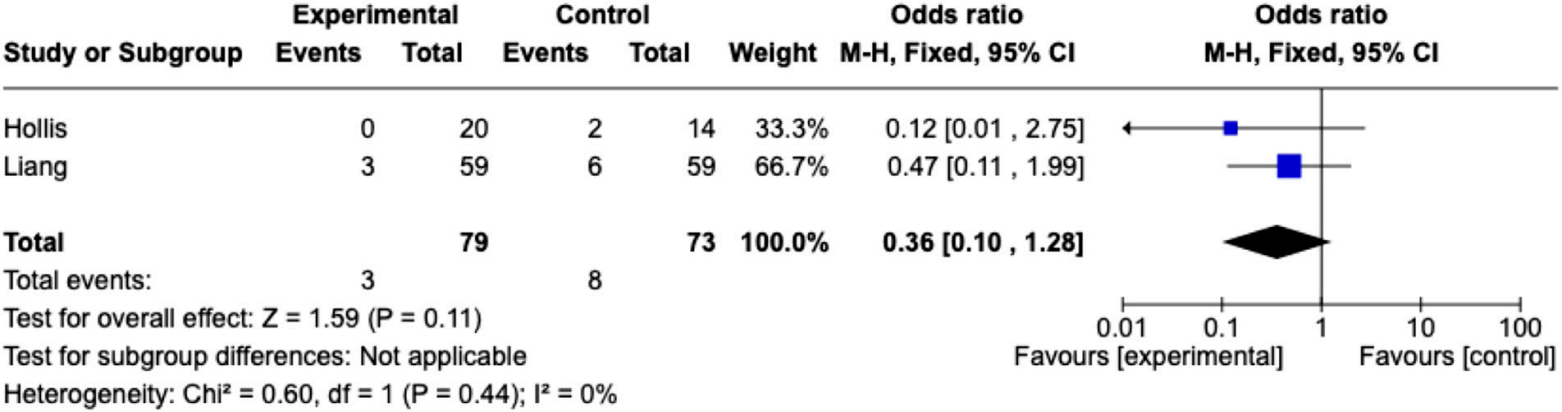
Meta-analysis of wound complications.

Meta-analysis for weight loss was possible for two studies, although both also contained patients undergoing non-hernia surgery [14, 15] (Figures 2, 3). This did not demonstrate a significant reduction in weight (meta-analysed weight loss 3.64 kg (95% confidence interval −2.07 – 9.35; random effects model) or complications (odds ratio 0.36 [0.1–1.28]; fixed effects)

### Bias

**FIGURE 4:**
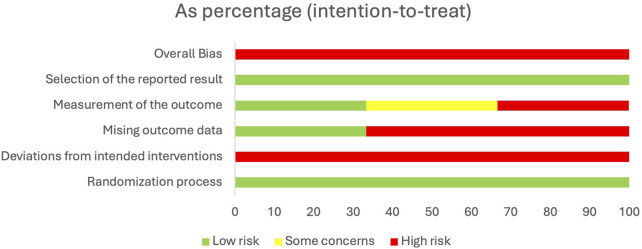
Assessment of bias.

Overall bias was rated as ‘High’ for all three studies and both outcomes (Figure 4). Risk of bias was mainly determined to be due to deviations from the intervention (i.e., differential drop out from pre-operative weight loss intervention compared with usual care), missing data, and how these were assessed and reflected within the results.

### Certainty of Effect

Due to the high risk of bias, combined with study heterogeneity and small number of studies, certainty of effect was determined as “very low” for both meta-analyses (Table 2).

**TABLE 2:** GRADE evidence table.

GRADE summary of findings
People: Obese adults Settings: Awaiting elective hernia repair Intervention: Any weight reduction intervention Comparison: Standard of care

Outcomes	Effect (95% CI)	Number of studies	Certainty of the evidence (GRADE)^a^	Comments
Reduction in body weight (kg)	3.64 (−2.07 – 9.35)	2	⊕⊖⊖⊖ Very low	Based on study quality and considerable study and intervention heterogeneity
Surgical complications	0.36 (0.10–1.28	2	⊕⊖⊖⊖ Very low	Based on study quality and considerable study, intervention and outcome heterogeneity

^a^ GRADE, working group grades of evidence. High = This research provides a very good indication of the likely effect. The likelihood that the effect will be substantially different^‡^ is low.

Moderate = This research provides a good indication of the likely effect. The likelihood that the effect will be substantially different^‡^ is moderate.

Low = This research provides some indication of the likely effect. However, the likelihood that it will be substantially different^‡^ is high.

Very low = This research does not provide a reliable indication of the likely effect. The likelihood that the effect will be substantially different^‡^ is very high.

^b^ Substantially different = a large enough difference that it might affect a decision.

## Discussion

Patients with obesity are more likely to develop hernias (particularly incisional hernias), and experience complications and hernia recurrence after surgery [6]. Weight is therefore considered a major modifiable risk factor before many forms of elective surgery, including hernia surgery. However, despite its importance there is no specific guidance before hernia surgery, other than a recommendation to lose weight [4]. We undertook the first dedicated systematic review of which we are aware, to identify and appraise the evidence for weight loss before hernia surgery derived from RCTs.

We identified 4 reports derived from 3 RCTs. One described a multidisciplinary intensive intervention based on dietetics and psychology; two described dietician-led VLCDs. Overall, at individual study level prehabilitation was variably associated with weight loss and complications. Both RCTs of VLCD demonstrated feasibility and significant weight loss; the more complex intervention did not, but this might be explained by the intervention ending if patients met a pre-defined weight loss threshold. Neither VLCD RCT demonstrated a difference in complications; the more complex RCT initially reported a more patients in the intervention arm being hernia and complication-free at 30 days, however, this was not sustained at 2 years. We were able to undertake meta-analysis of absolute weight loss and wound complications for both studies using VLCD; both were smaller and included patients undergoing non-hernia surgery. We did not demonstrate a significant difference in either endpoint, with “very low” certainty of effect.

A limited number of conclusions can be drawn from these studies. Firstly, RCTs on this topic are feasible in terms of recruitment, acceptability and delivery. Secondly these interventions seem well tolerated, and effect significantly greater weight and fat loss than their controls, potentially without lean body mass loss. However, whether these interventions translate into better surgical outcomes is unclear. Neither RCT using a VLCD found a difference in complications, although this is not unexpected when considering the surgical heterogeneity of the group and the fact that neither study was powered for these secondary endpoints. Liang et al, in the largest and hernia-specific RCT found more patients to be hernia and complication-free at 30 days using a composite metric, but this effect did not persist at 2 years [12, 13]. This may be due to the difficulties in using a composite metric, but also the fact that hernia-recurrence is extremely rare within 30 days of surgery, but relatively common within 2 years and can be related to a considerable number of factors (such as hernia and repair techniques) which may not be controlled within this study.

There remain a number of important and thus far unanswered questions. Firstly, whether pre-operative weight loss translates into better outcomes of surgery, in terms of complications, hernia recurrence and quality of life. Secondly, how this is best achieved (i.e., by what intervention; VLCD, advice and support, pharmacological, or a combination). Thirdly, how long this should be performed for and to what target. And fourthly, what effects these interventions may have on body composition, hernias and their repair. And finally, how this is best integrated within collaborative patient-centred decision-making [16].

The included RCTs, as with practice anecdotally, used understandably arbitrary weight loss thresholds (for example, 5 or 7%). However, as the impact of weight on recurrence and complications is non-linear, it seems likely that greater weight loss is more important in patients with more weight to lose (i.e., perhaps targets should be higher for those at a higher weight). Furthermore, some patients (for example, those with hernias at risk of loss of domain) may require substantial weight loss to make surgery technically feasible. It is unclear why efficacy varies for patients, and also whether weight loss is linear within these interventions; if not, however, it seems likely that prehabilitation may follow a law of diminishing returns, in that there may exist an inflection point whereby further attempts at weight loss (and therefore delay to surgery) are associated with diminishing returns (i.e., less additional weight loss), and perhaps an inflection point of negative returns (whereby further delay actually results in worse outcomes). This could occur due to hernias enlarging (thereby requiring larger, riskier and less successful surgery), and complications such as bowel obstruction or strangulation within the hernia. It is also unclear how interventions resulting in weight loss differ in their effects on body composition (visceral and subcutaneous fat, and lean body mass), and how these effect the technical and functional results of surgery.

The conclusions of this review are limited mainly by the lack, heterogeneity and bias of the included evidence, as well as the inclusion of a small number of patients undergoing non-hernia surgery. Our search term was focussed, and therefore it may have failed to identify potentially relevant studies. Meta-analysis was similarly limited by this heterogeneity.

In summary, despite its potential importance there remains a lack of high quality randomized evidence for the efficacy of pre-operative weight loss interventions before hernia surgery, and their impact upon complications. There is therefore a clear need for high quality large RCTs to firstly determine whether there is benefit to weight loss before elective hernia surgery, and secondly compare interventions to determine how best to achieve this. In the absence of such RCTs, prehabilitation before surgery should be personalised and based on collaborative patient-centred decision-making, taking into account the complex range of inter-related factors affecting patients’ weight, their hernias, the technical aspects of repair, and the practicality of delivering this within the resources available [16].

## Footnotes

1
http://pubmed.ncbi.nlm.nih.gov

2
https://www.embase.com/

3
https://www.cochranelibrary.com/central
